# Understanding of Stress‐Driven Internal Short Circuit Mechanisms in Lithium‐Ion Batteries with High SOCs

**DOI:** 10.1002/advs.202302496

**Published:** 2023-08-09

**Authors:** Xudong Duan, Jiani Li, Yikai Jia, Xiang Gao, Lubing Wang, Jun Xu

**Affiliations:** ^1^ Department of Automotive Engineering School of Transportation Science and Engineering Beihang University Beijing 100191 China; ^2^ Department of Mechanical Engineering and Engineering Science The University of North Carolina at Charlotte Charlotte NC 28223 USA; ^3^ Vehicle Energy & Safety Laboratory (VESL) North Carolina Motorsports and Automotive Research Center The University of North Carolina at Charlotte Charlotte NC 28223 USA; ^4^ School of Civil Aviation Northwestern Polytechnical University Xi'an Shaanxi 710072 China; ^5^ Key Laboratory Impact & Safety Engineering Ministry of Education Ningbo University Ningbo Zhejiang 315211 China; ^6^ School of Data Science North Carolina Motorsports and Automotive Research Center The University of North Carolina at Charlotte Charlotte NC 28223 USA

**Keywords:** battery safety, internal short‐circuit, lithium‐ion batteries, multiphysics modeling, soc effect

## Abstract

The characteristics of internal short circuits (ISC) play a critical role in determining the thermal runaway behaviors and associated hazards of lithium‐ion batteries (LIBs). However, due to safety concerns and limitations in *operando* characterization at high state‐of‐charges (SoCs), the fundamental understanding of stress‐driven ISCs under high SOC situations (above 30%) is still lacking. In this study, combined post‐mortem characterization and multiphysics modeling is employed to clarify the evolution of ISC modes in LIBs with high SOCs. These findings reveal that the triggered ISC mode is SOC‐dependent, with the Al current collector (Al)‐Anode coating (An) mode dominant in high SOC situations. Experimentally obtained ISC resistance for the specified ISC mode is then assigned to the corresponding ISC region in the established multiphysics model, allowing for accurate coupling of the electromechanical relationship and prediction of mechanical‐electrical‐thermal responses of the LIB. Finally, a simple yet effective approach is proposed for avoiding the Al‐An mode after battery fractures, achieved through surface notches on electrodes. Results discover novel phenomena for ISC in high SOC cells and reveal the underlying mechanisms, highlighting the importance and potential of battery structural design for developing next‐generation robust batteries.

## Graphical abstract

Stress‐driven ISCs of lithium‐ion batteries under high SOC situations is experimentally investigated where the Al collector (Al)‐Anode coating (An) mode is dominant. Based on the multiphysics model, the deformation state of internal components and other parameters such as the temperature are revealed. Finally, the model is used to explore how to avoid the Al‐An mode after battery fractures.

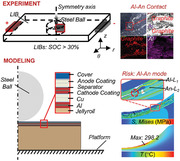

## 1 Introduction

With the expanding market of electric vehicles (EVs), the fires/explosion accidents caused by lithium‐ion batteries (LIBs) in EVs demonstrate a significant increase in both statistical data and news headlines,^[^ ^1^ ^]^ which hinders the further development of EVs. Safety issues caused by mechanical abusive loading are of great interest to both academia and industries. Internal short‐circuit (ISC), and thermal runaway (TR) of LIBs (single cell, module, or pack) are two major safety‐related events that have attracted highlighted focuses.^[^ ^1d,c^ ^]^

The ISC is generally considered the initial event followed by possible TR (in some extreme cases, we will have fires/explosions) under mechanical abusive conditions.^[^ ^2^ ^]^ The mechanism of the ISC is only partially understood, and how to mitigate or diminish the safety issues have become a fuzzy problem. Experimentally, compression,^[^ ^3^ ^]^ indentation,^[^ ^3^, ^4^ ^]^ bending,^[^ ^3^, ^5^ ^]^ and penetration,^[^ ^6^ ^]^ serving as the typical mechanical abusive conditions, were selected to trigger the external stress‐driven ISCs. The multiphysics responses of the LIBs, including the load *(F)*, displacement (*d*), voltage (*U*), and temperature (*T*) were obtained and analyzed. Further, the internal contacts among components of LIBs (i.e., the battery cover, separator, cathode (consisting of the cathode coating and Aluminum foil), and anode (consisting of the anode coating and Copper foil) under different loadings were investigated. First, the contact between each part of the cathode and anode was classified into four ISC modes, including the cathode coating‐anode coating (Ca‐An) mode, cathode coating‐Copper foil (Ca‐Cu) mode, Aluminum foil‐anode coating (Al‐An) mode, and Aluminum foil ‐Copper foil (Al‐Cu) mode.^[^ ^7^ ^]^ These four ISC modes produced different ISC resistances (*R*_ISC_), dominating the follow‐up thermal runaway behaviors.^[^ ^8^ ^]^ For the thermal responses, the Al‐An mode owned the highest power generated‐time curve (among four ISC modes) and was considered the most dangerous ISC mode.^[^ ^1^, ^7^ ^]^

To investigate the ISC mode of LIBs deformed, a common method is the post‐mortem examination, where the LIBs were disassembled after being loaded.^[^ ^9^ ^]^ However, once the LIBs experienced a large deformation/fracture, the internal components became difficult to separate in local zones, and the disassembling could cause unavoidable artificial damage. Thus, nondestructive testing, such as X‐ray computed tomography (CT) was employed to observe the internal structural change of LIBs,^[^ ^9^, ^10^ ^]^ and the debonding of anode coating was captured.^[^ ^9a^ ^]^ However, due to the spatial resolution limitation of CT, the separator and foils with an extremely small thickness (a few µm) are generally difficult to capture. Also, distinguishing each layer from CT images became challenging.^[^ ^9a^ ^]^ In our previous research, combining the post‐mortem experiment and CT, we revealed the ISC mode evolution inside the LIBs with 10% SOC under various loadings.^[^ ^9a^ ^]^ However, the mechanism of stress‐driven ISCs under high SOC situations (≥ 30%) is still unclear. Especially after the load (*F*) drops sharply, the voltage (*U*) of LIBs with high SOCs does not drop to 0 but remains at a high value (close to the initial voltage). In contrast, the *U* of LIBs with low SOCs would immediately drop to 0,^[^ ^9^, ^11^ ^]^ indicating that the underlying mechanism for the electrochemical behaviors is fundamentally different in high SOC scenarios.

To supplement the deficiency and together reveal a more intrinsic understanding of *operando* observation, physics‐based computational modeling is a critical way to solve the problem. The finite element model (FEM) has been proven to provide a quantitative picture of the behaviors and phenomena inside the batteries. For computational modeling, first of all, a coarse or detailed description of LIBs was developed to predict the mechanical response of LIBs in the mechanical model.^[^ ^3^, ^12^ ^]^ Further, to bridge the electrochemical and mechanical behaviors, the stress‐ and strain‐based ISC criteria^[^ ^13^ ^]^ were developed. Some researchers determined the strain/stress of the separator at the critical point when the experimental *F* or *U* dropped, as the failure of the separator is the direct reason for triggering the ISC.^[^ ^9^, ^12^, ^14^ ^]^ Then, a multiphysics model was built to predict the subsequent electrothermal responses of LIBs,^[^ ^9^, ^14^ ^]^ but lack of a description of the actual contact among components. Based on detailed or locally detailed mechanical models, the contact between the cathode and anode inside the LIB under different loadings was accurately predicted.^[^ ^9^, ^15^ ^]^ Upon the ISC triggering criteria, follow‐up submodels, e.g., ISC model, thermal model, TR model, *etc*. are used to describe the multiphysical behaviors. However, one critical parameter, i.e., ISC resistance, *R*_ISC_ is usually determined empirically: *R*_ISC_ of the ISC mode was not directly measured by experiments but calibrated based on the experimental *U–d* curves.^[^ ^11^ ^]^ Since *R*_ISC_ dominates the thermal generated during the ISC, governs the subsequent side reactions, and significantly influences the TR hazards, it is imperative to understand the underlying determinant factors of *R*_ISC._ Recently, the machine‐learning methodology has provided a new way to conduct the computational modeling and prediction without an in‐depth understanding of certain parameters to predict the safety risks of LIBs based on the dataset (including *F*, *d*, *U*, and *T*) of battery failure tests.^[^ ^13^, ^16^ ^]^ However, without domain knowledge, the model is usually limited and not generalized enough for various engineering applications.

In this study, first, we revealed the stress‐driven ISC mode evolution of LIBs with high SOCs (≥ 30%) upon the mechanical abusive loading experimentally. Then, the ISC mode was confirmed based on the element distribution analysis in the section through the Energy Dispersive Spectroscopy (EDS) technology. Besides, a locally refined mechanical model was established and can accurately predict the ISC behaviors under different loadings proved by experiments. Based on the experimental *R*_ISC_‐stress curves, the *R*_ISC_ was carefully characterized and applied in the multiphysics model to enable accurate predicting of the electrothermal responses of the LIBs under various loadings. Finally, a possible and engineering‐oriented design to mitigate ISC consequences is also provided.

## 2 Results

**Figure 1:**
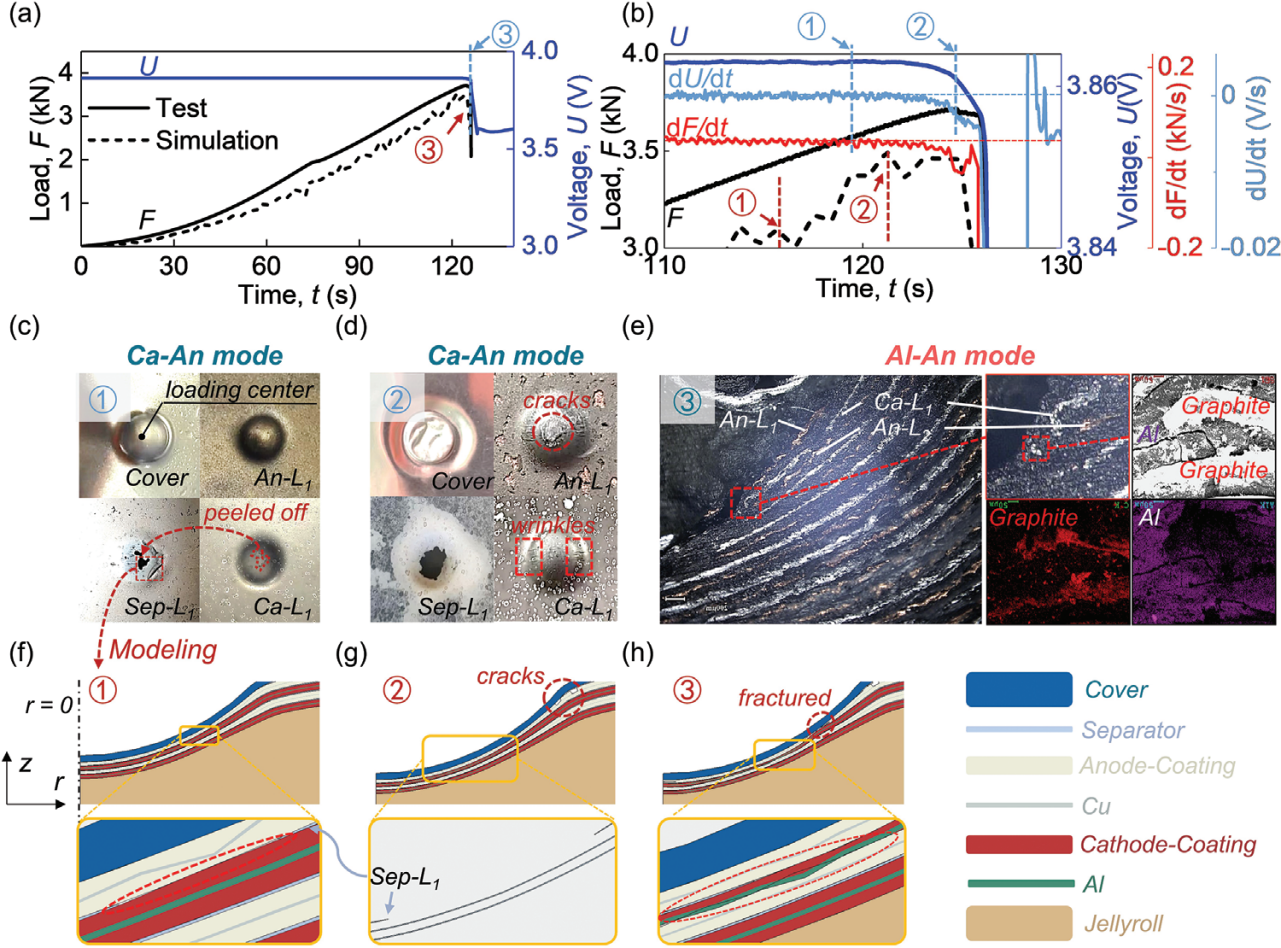
Typical results of the LIB with 60% SOC under 4mm‐*R*_ball_ indentation condition. a) Load (*F*)‐, and voltage (*U*)‐time (*t*) curves from the indentation test and the *F–t* curve from the 2D Mechanical Model; b) load (*F*)‐, voltage (*U*)‐, load change (*dF/dt*)‐, and voltage change (*dU/dt*)‐time (*t*) curves from the indentation test and the *F–t* curve from the 2D Mechanical Model within the *t* from 110s to 130s; failure morphology of LIBs after being loaded until c) Point 1, d) Point 2, and e) Point 3 based on experiments; and the failure morphology of LIBs after being loaded until f) Point 1, g) Point 2, and h) Point 3 based on the 2D Mechanical Model.

To investigate the electrochemical and mechanical failure of the cells (load‐drop exceeding 40% of the maximum value) with high SOCs in a repeatable and controllable manner, we used a steel ball with a radius of 4 mm (*R*_ball_ = 4 mm) to indent LIBs^[^ ^9a^ ^]^ with 60% SOC at a speed of 1 mm min^−1^ until the battery is electrochemically failed (**Figure** **1**a). Note that LIBs with 60% SOC did not experience TR (explosion/fire) after failure. Thus, it is feasible to study the internal structure in a post‐mortem manner while still exhibiting significantly different behaviors compared to the counterparts in low SOC cases, serving as a perfect candidate to investigate cases with high SOC (≥30%). Besides, a locally detailed 2D axisymmetric mechanical model was established ( Section 5) to predict the mechanical response of the battery (Figure 1a) where the triggering of ISCs can be understood.

### 2.1 Triggering and Evolution of Cathode Coating‐Anode Coating (Ca‐An) Mode

At Point 1 (*t* = 118s), the Ca‐An mode was triggered. The voltage (*U*) began to drop, and the d*U*/d*t* started to become smaller than 0 (unrecoverable) (Figure 1b), indicating that an ISC occurred. Additionally, the load (*F*) did not drop, but the d*F*/d*t*‐*t* curve began to decline, exhibiting a “load softening” phenomenon (Figure 1b), usually attributed to the intrusion among coatings. The separator failure was dominant currently.^[^ ^9a^ ^]^ To further verify, the sample was disassembled after being loaded to Point 1 (Figure 1c). The 1^st^‐layer of cathode coating (Ca‐L_1_) and anode coating (An‐L_1_) were generally intact, protecting the foils from exposure. After disassembly, the residual part of the Ca‐L_1_ on the 1st ‐layer separator (Sep‐L_1_) indicated that the coatings could come into contact through the separator, triggering the Ca‐An mode. Note that the part far away from the loading center of Ca‐L_1_ was more easily peeled off and adhered to the Sep‐L_1_ than the part right at the loading center (Figure 1c), consistent with our previous research.^[^ ^9a^ ^]^

Based on the 2D mechanical model calculated to 116s (Point 1, Figure 1f), the Sep‐L_1_ starts to fracture at *r* (position at *r* axis) = ≈1.83 mm (not at the loading center where *r* = 0), which proves that the stress in Sep‐L_1_ first reaches the maximum along the craters of the loading center under indentation. Meanwhile, the electrode coatings remain intact and contact each other at the position where the Sep‐L_1_ fractures, triggering the Ca‐An mode.

Until Point 2, the *F* suddenly decreased slightly (Figure 1b). First, the d*U*/d*t* curve demonstrated a smooth transition at Point 2 (Figure 1b), indicating no other ISC modes (with higher ISC conductivity than the Ca‐An mode^[^ ^8^ ^]^) occurred. Further, by disassembling the battery loaded to Point 2, the cover remained complete, and the foils were still covered by coatings (Figure 1d), which proved the Ca‐An mode at Point 2. Due to the loading increase, the coating area adhered to the Sep‐L_1_ became larger than that at Point 1 (Figure 1d). It caused more serious Ca‐An type ISC (even under the same ISC mode), demonstrated in the larger absolute value of d*U*/d*t* (Figure 1b). The slight drop of *F* was usually related to the failure of coatings.^[^ ^9a,b^ ^]^ The center region of An‐L_1_ dislocated from the surrounding region to form cracks, and Ca‐L_1_ showed wrinkles (Figure 1d), which could contribute to the slight drop of *F*.

At Point 2, the simulated *F–t* curve starts to enter a platform showing a similar trend with the test (Figure 1b). Some cracks appear in the An‐L_1_ as well. The Ca‐L_1_ becomes much thinner than at Point 1 (Figure 1g), which may lead to a platform‐like load response. Despite cracks in the coating, the foils are still well protected from contact between electrodes (Figure 1g), suggesting the battery is still under the Ca‐An mode. The failure region of the Sep‐L_1_ extends from *r* = 0.83 mm to *r* = 2.19 mm, and its area expands to ≈4 times as much as that at Point 1, responsible for more serious ISC. Compared with the ISC location at Point 1 (from *r* = 1.83 mm to *r* = 2.06 mm), the ISC region gradually evolves toward the loading center with the load increase. It can be supported by the residual coating in the Sep‐L_1_ at Point 2 where more coating remained near the loading center of Sep‐L_1_ than that at Point 1 (Figure 1d).

### 2.2 Triggering of Aluminum Foil‐Anode Coating (Al‐An) Mode

Until Point 3, the *F* and *U* dropped sharply, and the d*U*/d*t‐t* curve showed an obvious inflection (Figure 1a,b), indicating a new ISC mode. For further proof, the battery loaded until Point 3 was wrapped with epoxy resin for shape fixing. Then, the optical microscope exposed the sample section under the steel ball by wire‐electrode cutting for post‐mortem observation. The 1^st^‐layer anode fractured, and the remaining part (connected with the negative tab and marked as An‐Main) did not come into contact with the Al of 1^st^ layer cathode (Al‐L_1_) (Figure 1e). It declares that the Al‐Cu (copper foil) mode was not triggered. The Al‐L_1_ was probably squeezed into the geometric area of the 2^nd^ layer anode (Figure 1e). We used the Energy Dispersive Spectroscopy (EDS) analysis to provide direct evidence. Results showed that the metal Al was surrounded by graphite (Figure 1e). It declares that Al‐L_1_ was pressed into the coating of 2^nd^ layer anode (An‐L_2_), which belonged to the An‐Main, thus forming a stable Al‐An ISC mode. Note that the part of cathodes connected with tabs (Ca‐Main) contacting with the An‐Main can lead to the stable ISC (illustrated as the irreversible decline of *U*). After being connected, the fractured part (separated from the Ca‐Main/An‐Main) has limited charges and cannot form a stable circuit. After the battery fails, the contact between Ca‐Main and An‐Main causes the continuous heat source to induce subsequent thermal runaway. It deserves more attention than the connection among fractured parts. This discussion becomes essential when comparing the failure morphology of different models with electrodes pre‐notched after the battery fails in the Section 3.2.

At Point 3 (Figure 1h), the 1^st^ layer anode fractures and cracks into two parts. Due to the space left after fracture, the 1^st^ layer cathode squeezes upward, causing a large deformation. The Ca‐L_1_ becomes much thinner than that at Point 2 and fails from *r* = 1.3 mm to *r* = 1.68 mm. Such phenomena may result in the exposure of the Al‐L_1_. The An‐L_2_ remains relatively complete, leading to the Al‐An mode.

### 2.3 Ex Situ Multiphysics Behavior Evolutions upon Ball‐Indentation

LIBs were loaded to each critical point (Points 1–3), and the *U* and temperature (*T*) of LIBs remained recorded after samples were unloaded. Then, the effect of ISCs (electrothermal response of LIBs) triggered by different stresses was experimentally investigated. A 2D axisymmetric multiphysics model is established (including the mechanical, ISC, battery, heat, and thermal runaway modules (Section 5)). The multiphysics model will be validated by experiment and also assist in unraveling the mechanical‐electrical‐thermal coupling mechanism.

#### 2.3.1 Point 1

**Figure 2:**
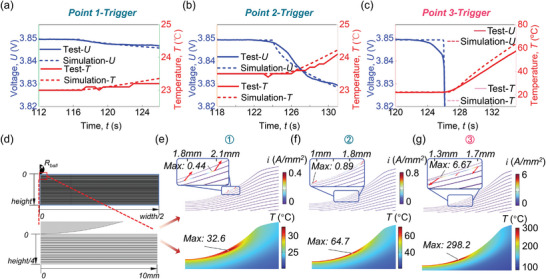
The *U*‐, and temperature (*T*)‐*t* curves within 10s after LIBs were loaded to a) Point 1; b) Point 2; c) Point 3 from the experiment and 2D Multiphysics Model; d) Schematics of the display range for subsequent ISC region of the battery; the current density and temperature distribution after LIBs were loaded to e) Point 1; f) Point 2; g) Point 3 computed by 2D Multiphysics Model.

Within 10s after the LIB was loaded to Point 1 (and unloaded), *U* only decreased by ≈3.4 mV with *T* increasing by 0.2 °C (**Figure** **2**a). Even if the ISC lasted for 120s, *U* only decreased by ≈13.7 mV, with *T* increasing by 1.6 °C (Figure S6b, Supporting Information). The Ca‐An mode triggered at Point 1 demonstrates a limited effect (subtle change of *U* and *T*).

For the mechanical‐electrical coupling mechanism, the ISC location at Point 1 (from *r* = 1.83 mm to *r* = 2.06 mm) is accurately predicted by the multiphysics computational model and then set in the ISC module (Figure S3a,b, Supporting Information). The ISC conductivity κ_*ISC*_ for the ISC region can be obtained by the law of resistance:

(1) $$R_{\mathrm{ISC}}=\frac{L}{\kappa_{\mathrm{ISC}}S}$$

where *R*_ISC_ is the ISC resistance and *L* and *S* are the height and sectional area of the ISC region, respectively. The areal resistance *R*_ISC_ · *S* of each ISC mode under different stresses was tested previously^[^ ^17^ ^]^ (Figure S3e, Supporting Information). At Point 1, the pressure between Ca‐L_1_ and An‐L_1_ is estimated as ≈100 MPa based on the computational model (Figure S3d, Supporting Information), and *R*_ISC_ · *S*≈12π(Ωmm^2^). Then, according to the ISC region after deformation (determining the *L* and *S*), κ_ISC_can be obtained as ≈1.53(S m^−1^) (Equation 1). The current inside the LIB (*I*) flows from the Ca‐L_1_ to the An‐L_1_ with 0.44A mm^−2^ as the maximum current density (*i*) (Figure 2e). The total *I* reaches ≈0.49 A by integrating *i* on the boundary of the separator. The LIB module takes the *I* as the boundary condition and outputs the *U* evolution, agreeing well with the test (Figure 2a).

Meanwhile, the joule heat triggered by *R*_ISC_ and the LIB internal resistance (*R_cell_*) serve as the heat source in the heat module. The temperature (*T*) distribution of the LIB was measured to be 32.6 °C as the maximum within 10s after the ISC was triggered (Figure 2e). The *T* at measuring points (Section 5) of the LIB can also be predicted accurately (Figure 2a).

#### 2.3.2 Point 2

At Point 2, *U* decreased by ≈20 mV, with *T* increasing by 0.6 °C (Figure 2b). After 60s since the ISC was triggered, the *U* decreased by ≈43.9 mV, with *T* increasing by 5.4 °C (Figure S6c, Supporting Information). The Ca‐An mode evolving to Point 2 demonstrates a more serious ISC degree than at Point 1 (initiation).

From Points 1 to 2 in the computational model, the failure area of the Sep‐L_1_ evolves to the more aggressive Ca‐An mode (Section 2). The ISC region, whose area is ≈4 times that at Point 1 (from the 2D mechanical model) is set in the ISC module (Section 5). The pressure between Ca‐L_1_ and An‐L_1_ reaches ≈130 MPa and *R_ISC_* · *S* is obtained as 10.44π(Ωmm^2^) (Figure S3e, Supporting Information). Then, κ_*ISC*_ for the ISC region at Point 2 can be obtained as 3.07 (S m^−1^) (Equation 1). The maximum *i* (*i*_max_) reaches 0.89A mm^−2^ (Figure 2f), and the total *I* reaches ≈2A. The *U–t* curve calculated by the LIB module can also match well with the test (Figure 2b). Besides, the *T–t* curve can also be predicted accurately (Figure 2b). Notably, within 10s after Point 2, based on the heat module, the maximum temperature of the LIB (*T*_max_) reaches 64.7 °C (Figure 2e). For the LIBs with Li‐plating, this ISC degree brings the risk of thermal runaway (TR) because the plated Li can lower the threshold value of triggering *T* for the TR.^[^ ^18^ ^]^

#### 2.3.3 Point 3

When the LIB was loaded to Point 3, the voltage‐drop and temperature‐rise speed became significantly fast (Figure 2c). Within 10s, the *U* decreased by ≈0.2 V (Figure S6a, Supporting Information) with *T* increasing by ≈36 °C (Figure 2c). When the Al‐An mode had been initiated for 60s inside the LIB with 60% SOC, the *T* at measuring points reached its maximum ≈136 °C (Figure S6a, Supporting Information). The Al‐An mode dominated the electrothermal responses of the LIB when the Ca‐An mode still existed.

The Al‐An ISC region (from *r* = 1.3 mm to *r* = 1.68 mm) is set between the Ca‐L_1_ and An‐L_2_ based on the 2D Mechanical Model (Figure S3b, Supporting Information). The pressure between Al‐L_1_ and An‐L_2_ is estimated as ≈140 MPa, and *R_ISC_* · *S* of Al‐An mode is obtained as 0.57π(Ωmm^2^) (Figure S3f, Supporting Information). Then, κ_*ISC*_ can be obtained as (Equation 1). The *I* flows from Ca‐L_1_ to the An‐L_2_ with i_max_ reaching 6.67A mm^−2^ (Figure 2g). The total I reaches ≈8.37 A causing the U evolution to match well with the test (Figure 2c). Furthermore, the *T*–*t* curve from the heat module can be predicted accurately (Figure 2c).

Under the Al‐An mode, besides the joule heat, thermal runaway (TR) reactions (i.e., SEI decomposition, anode‐electrolyte reaction, and cathode‐electrolyte reaction) can also be triggered and produce heat sources by the thermal runaway module (Section 5). The *T*_max_ reaches 298.2 °C within 10s after Point 3 (Figure 2g). With the increase of SOC, the enthalpy of TR reactions will increase, causing more heat sources,^[^ ^8^, ^19^ ^]^ greatly increasing the risk of TR (fire/explosion).

## 3 Discussion

### 3.1 SOC Effect on the ISC Mode of LIBs after Mechanical Failures

The effect of stress‐driven ISC after the mechanical failure of the LIB (load drop exceeds 40% of the maximum value) is the most serious and worthy of attention. Aiming at this stress level, we will discuss the SOC effect on the ISC mode by conducting steel ball indentation tests on LIBs with different SOCs (0%, 30%, 60%, and 80%) based on experiments.

**Figure 3:**
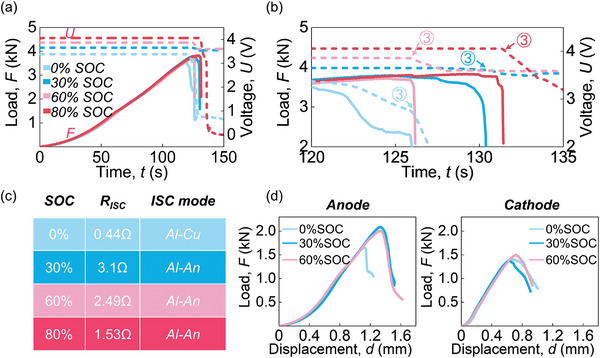
SOC effect on the LIBs and electrodes under the steel ball indentation. a) *F‐*, and *U–t* curves of LIBs with different SOCs; b) *F‐*, and *U–t* curves of LIBs with different SOCs within the *t* from 120 to 135s; c) the *R*_ISC_ and ISC mode after battery failure under different SOC situations; and d) *F–d* curves of the anode and cathode with different SOCs.

The *F*–*t* curves of different SOCs (0–80%) for the mechanical response were highly similar. They only showed differences near the battery failure, reflected in the maximum *F* (*F*_max_) and *t* (**Figure** **3**a). *U* under 0% SOC situation after battery failure could reach 0, while *U* under 30% and 60% SOC situations decreased moderately and remained at a high value (≈3.5 V) (Figure 3a). In the 80% SOC situation, *U* did not immediately drop to 0 after failure but to ≈3 V within ≈5s (Figure 3b). At this moment, *T* rose to over 200 °C (Figure S7c,d, Supporting Information). The fires and explosion (TR) caused structural damage to samples causing the *U* to drop sharply (to ≈0 V) (Figure S7d, Supporting Information). Further, by analyzing the slope of *U–t* curves after Point 3 (mechanical failure point) (Figure 3b), the slope under 0% SOC situation was significantly greater than the slopes under high SOC situations (SOC≥ 30%). The slope under each high SOC situation (30%–80%) was relatively close to each other (Figure 3b).

The *R*_ISC_ after Point 3 under each SOC situation was roughly estimated by:^[^ ^9c^ ^]^

(2) $$\Delta U=\frac{U\times R_{\mathrm{cell}}}{R_{\mathrm{cell}}+R_{\mathrm{ISC}}}$$

where Δ*U* was defined as the *U* decline within 0.5s after Point 3, which contributed to characterizing the slope of *U–t* curves intuitively. The *R*_ISC_ of 0% SOC reached 0.44 Ω while the *R*_ISC_ of each high SOC was in the order of ≈1 Ω (Figure 3c). Our previous study revealed that the Al‐Cu (copper foil) mode occurred after the mechanical failure of 10% SOC LIBs.^[^ ^9a^ ^]^ This study determined the Al‐An mode after the failure of 60% SOC LIBs. Given that the electrical conductivity ranks as Cu > Al > An > Ca, the *R*_ISC_ in low SOC cases can also be considered as the outcome of the Al‐Cu mode (similar to the 10% SOC situation) (Figure 3c). Since the *R*_ISC_ of each high SOC (30%–80%) was similar, it then becomes reasonable to believe that the Al‐An mode will be triggered after the failure of LIBs under relatively high SOCs (≥ 30%). Besides, similar results regarding SOC independence on the *R*_ISC_ of Al‐An mode,^[^ ^20^ ^]^ are ≈1 Ω,^[^ ^8^ ^]^ supporting our hypothesis. Note that the differences in *R*_ISC_ under high SOC situations (Figure 3c) may be due to the different local roughness and mechanical stress (related to the contact resistance^[^ ^21^ ^]^) inside LIBs with different SOCs.

To further explore the mechanism of the SOC effect on the ISC mode after battery failure, the steel ball indentation tests on electrodes with different SOCs were carried out (Section 5) (Figure 3d). The *F–d* (displacement) curves of the cathode were close to each other, demonstrating little SOC effect. For the anode, the *F–d* curves were similar before they descended. The SOC mainly affected the failure behavior of the anode. The *F*_max_ of 30% and 60% SOC situations were close to each other and higher than the *F*_max_ of 0% SOC declaring the enhancement of the mechanical properties of the anode coating due to the insertion of Li‐ions.^[^ ^9^, ^22^ ^]^ For the LIBs with SOC larger than 30%, the anode coating will be more difficult to be squeezed to failure and can protect the Cu from exposure after battery failure. Besides, compared with *F–d* curves of the anode, the cathode under different SOCs all owned smaller *F*_max_ showing a weaker ability to resist the extrusion of the steel ball. The Al can be easy to exposure and participate in an ISC mode after failure of LIBs with different SOCs. Then, it becomes natural to expect the ISC mode to change from Al‐Cu mode to Al‐An mode with increased SOC (after battery failure). Note that 80% SOC LIBs were prone to explode and jet flames. For safety, the electrodes were not disassembled from 80% SOC LIBs for tests.

In our recent study,^[^ ^9a^ ^]^ we have combined steel ball indentation experiments on 10% SOC LIBs and the multiphysics model to explore the size effect on ISC mode evolution. Under the indentation of steel balls with different sizes (*R*_ball_ = 4mm–12.5 mm), 10% SOC LIBs all experienced similar evolution sequence of Ca‐An mode, Ca‐Cu mode, and Al‐Cu mode. Interestingly, with the increase of *R*_ball_, the triggering indentation displacement of each ISC mode became larger. It would be more difficult to trigger the ISC, due to the less serious stress concentration of the battery indented by the larger steel ball. Besides, the larger *R*_ball_ caused a larger radius of the deformation region under the same loading displacement, which caused larger ISC region and enabled the voltage of the LIB to decline faster with the larger indenter size. Similarly, for the LIBs with high SOCs (30%), we believe that the LIBs indented by different steel balls will experience the same ISC sequence as the situation with *R*_ball_ = 4 mm, where includes Ca‐An mode and Al‐An mode. The ISCs will be delayed as the steel ball size increases. With the indentation displacement increasing, the larger the steel ball is, the faster the voltage of the LIB will drop.

### 3.2 A Problem‐Solving Strategy for the ISC Mode of LIBs after Mechanical Failures

Once the battery mechanically fails (load drop exceeds 40% of the maximum value), the fracture and mutual contact of electrodes will be inevitable. Surface pre‐notches on electrodes are expected to induce contact status between the cathode and anode, guiding to a milder ISC mode with a larger *R*_ISC_ than Al‐An mode, e.g., Ca‐Cu (≈10 Ω) or Ca‐An (≈100 Ω).^[^ ^8^ ^]^ Then, the risk of TR after battery failure can be greatly reduced.

**Figure 4:**
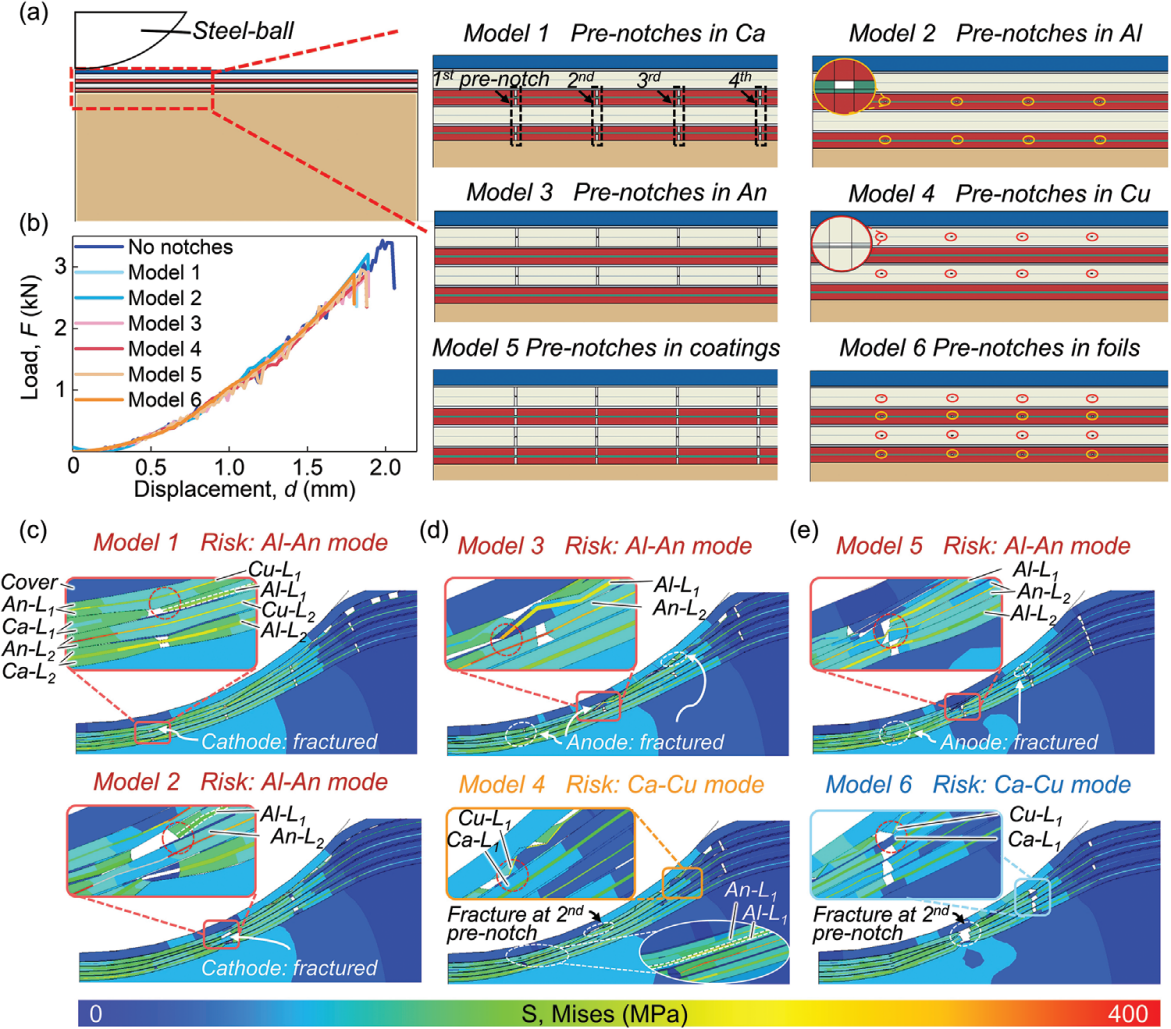
Surface pre‐notch effect on the ISC mode after battery failure. a) the arrangement of pre‐notches in six 2D Mechanical Models; b) the *F‐d* curves from 2D Mechanical Models; and the Von Mises Stress distribution after the battery mechanical failure computed by c) Models 1 and 2; d) Models 3 and 4; e) Models 5 and 6.

Since the surface pre‐notches can be set on coatings and foils, six 2D Mechanical Models with 4 surface pre‐notches set (named as the 1^st^–4^th^ pre‐notch respectively) are designed to discuss the effects of pre‐notches (**Figure** **4**a) (Section 5). The surface pre‐notches are set on the cathode coatings (Ca) in Model 1; on the cathode foils (Al) in Model 2; on the anode coatings (An) in Model 3; on the anode foils (Cu) in Model 4; on both cathode and anode coatings in Model 5 and on both Al and Cu foils in Model 6. The *F–d* curve of each model with surface pre‐notches coincides with the curve of the model without pre‐notches before battery failure (Figure 4b). The maximum displacement (*d*_max_) of each model with surface pre‐notches is close to each other and slightly less than the *d*_max_ of the model without pre‐notches (Figure 4b).

For the failure morphology after the *F–t* curve decreases sharply, in Model 1 with cathode coatings pre‐notched, the 1^st^ layer cathode fractured only at the 1^st^ pre‐notch (Figure 4c). The Al connected with Ca‐Main (the part of cathodes connected with the tab) is exposed and has the risk to contact with An‐L_2_ (Figure 4c), forming the stable Al‐An mode. In Model 2 with Al pre‐notched, the 1^st^ layer cathode fractures only at the 2^nd^ pre‐notch (Figure 4c). The exposed Al also has the risk to contact with An‐L_2_ (Figure 4c), forming the Al‐An mode. Comparing Models 1 and 2, the cathode with coating pre‐notched is more likely to fail in the region dominated by compressive stress near the 1^st^ pre‐notch. However, the cathode with foil pre‐notched is easier to fail first in the region dominated by shear stress near the 2^nd^ pre‐notch (Figure 4c). For the cathode pre‐notched situations, the cathode tends to fracture much earlier than the anode because the cathode is more prone to damage upon the extrusion of the steel ball than the anode (Figure 3d). At that time, the anode can maintain relatively intact. Then, the contact between exposed Al and relatively complete anode coating will inevitably form Al‐An mode.

In Model 3 with anode coatings pre‐notched, the 1^st^ layer anode fractures at the first three pre‐notches (Figure 4d). Therefore, only the part after 3^rd^ pre‐notch is connected with the An‐Main and can trigger stable ISCs. Fortunately, the cathodes remain complete near the 3^rd^ pre‐notch (Figure 4d), indicating no need to worry about serious ISCs. The 1^st^ layer cathode is squeezed in the gap of 1^st^ layer anode after the fracture at 2^nd^ pre‐notch (Figure 4d). The 1^st^ layer cathode experiences large deformation and fractures. The exposed Al has the risk to contact with An‐L_2_ (Figure 4d). The 2^nd^ layer anode only fractured at 1^st^ pre‐notch and remains relatively intact near 2^nd^ pre‐notch (Figure 4d). Then, the stable Al‐An mode can be triggered.

In Model 4, which uses pre‐notched Cu anode foils, the anodes are also difficult to fracture at the 1^st^ pre‐notch, similar to Model 2 with Al pre‐notched. Only the anode first layer breaks at the 2^nd^ and 3^rd^ pre‐notches. Additionally, the first layer cathode can be squeezed into the gap at the 2^nd^ pre‐notch of the 1^st^ layer anode. Since the anode coating is not removed in advance in Model 4, the gap is smaller than that in Model 3. The deformation of the first‐layer cathode is not sufficient to cause a fracture, but it fails cathode coatings (Figure 4d). The Al can contact the An‐L1, which is separated from the An‐Main, causing the Al‐An mode ISC with limited charges (ISC is unstable). Then, the risky region becomes the vicinity of the third pre‐notch where the exposed Cu‐L_1_ may contact the Ca‐L_1_, causing the stable Ca‐Cu mode.

In Model 5, where both cathode and anode coatings are pre‐notched, a layer‐by‐layer analysis shows that the risky region is focused on the vicinity of the 2^nd^ pre‐notch (Figure 4e). The An‐L_2_ has the risk of contacting both Al‐L_1_ and Al‐L_2_. However, the stable Al‐An mode can be triggered since the An‐L_2_ can still connect with the An‐Main at the 3^rd^ pre‐notch (Figure 4e).

In Model 6, where both Cu and Al foils are pre‐notched, the foils fracture easily, and the electrodes fracture neatly at the 2^nd^ pre‐notch (Figure 4e). Even if there is a risk of contact between Al foils and anode coatings at the 2^nd^ pre‐notch, it will not form a stable ISC since the anodes have fractured at the 3^rd^ pre‐notch (Figure 4e). The risky region is finally focused on the vicinity of the 3^rd^ pre‐notch, where the exposed Cu‐L_1_ has the risk of contacting Ca‐L_1_ to form a stable Ca‐Cu mode.

Comparing Model 4 and Model 6, which both have the risk of Ca‐Cu mode, the gap at the 2^nd^ pre‐notch in Model 6 is much more significant than in Model 4 (Figure 4d,e). The 1^st^ layer anode in Model 4 is more likely to contact the An‐Main at some point in the in‐plane direction than in Model 6. Therefore, the Al‐An contact at the 1^st^ pre‐notch (Figure 4d) may trigger a stable Al‐An mode in Model 4. Model 6, with both Al and Cu foils pre‐notched, ensures that the cathode and anode fracture at the 2^nd^ pre‐notch and separate from the Ca‐Main and An‐Main, respectively. Such design allows the risky region to become the vicinity of the 3^rd^ pre‐notch, where a mild stress concentration (≈60 MPa which is half of the Von Mises Stress around the 2^nd^ pre‐notch) exists (Figure 4e). The electrodes can remain relatively intact, reducing the risk of Al‐An mode more than in Model 4. Therefore, we believe that using Al and Cu foils with pre‐notches is the best arrangement to avoid Al‐An mode after battery failure.

### 3.3 Size and Distribution Effect of Surface Notches

#### 3.3.1 Size Effect

**Figure 5:**
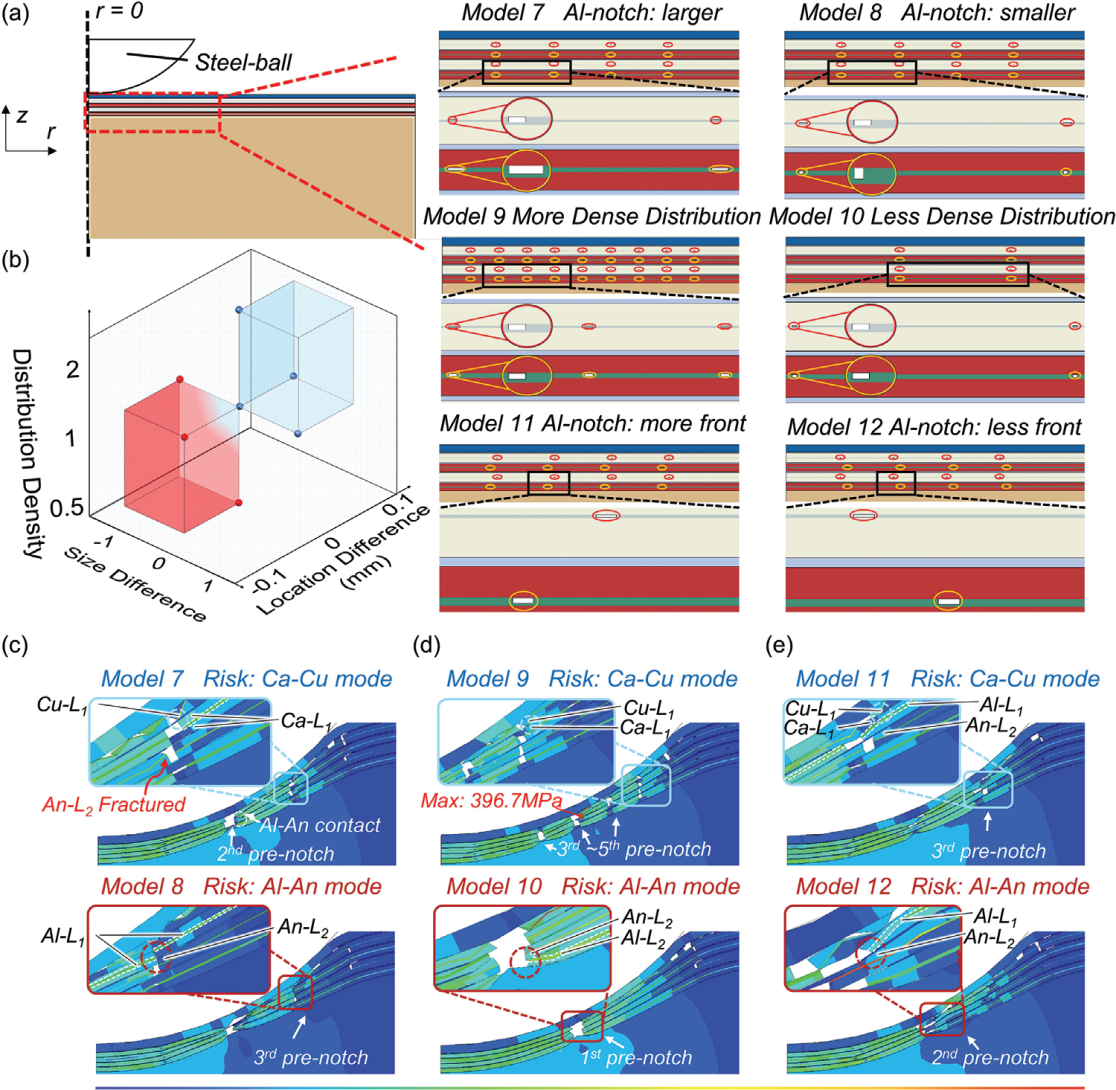
Parametric study on surface pre‐notches. a) the arrangement of pre‐notches in Models 7–12; b) the safety boundary of the LIB with pre‐notches after failure; and the Von Mises Stress distribution after the battery mechanical failure computed by c) Models 7 and 8; d) Models 9 and 10; e) Models 11 and 12.

When the width of pre‐notches (*w_notch_*) on Al and Cu keeps consistent and becomes half (Model S1) or twice (Model S2) that of Model 6 simultaneously (the size difference is marked as ‘0′), the possible ISC mode after battery failure is still the Ca‐Cu mode (Figure S11a, Supporting Information). For the situation with the *w_notch_* on Al and Cu inconsistent, two new models are established, i.e., the *w_notch_* of Al is twice that of Cu in Model 7 with the size difference marked as ‘1′ while half that of Cu in Model 8 (**Figure** **5**a) with the size difference marked as ‘−1′. Note that the *w_notch_* of Cu in Models 7 and 8 maintains the same as the *w_notch_* of Cu in Model 6.

In Model 7, the electrodes fracture at the 2^nd^ pre‐notch where the contact between Al‐L_1_ and An‐L_2_ occurs (Figure 5c). Since the anodes still fracture at the 3^rd^ pre‐notch (Figure 5c), similar to Model 6, it will not form a stable Al‐An mode. The risky region is also focused on the vicinity of 3^rd^ pre‐notch where the exposed Cu‐L_1_ has the risk of contacting Ca‐L_1_ to form the Ca‐Cu mode (Figure 5c). In Model 8, the vicinity of 3^rd^ pre‐notch can also be determined as the risky region for stable ISCs where the anodes and 1^st^ layer cathode fracture (Figure 5c). The parts of the 1^st^ layer cathode on both sides of the fracture are incredibly close and can contact each other under indentation (forming an electrical path). The dislocation of 1^st^ layer cathode causes the contact between Al‐L_1_ and An‐L_2,_ triggering the Al‐An mode.

Comparing Model 7 with Model 8, the Al with larger *w_notch_* becomes more easily and earlier to fracture, enabling the *F–d* curve of Model 7 to drop sharply earlier than that of Model 8 (Figure S11b, Supporting Information). The cell in Model 8 experiences larger indentation displacement. Then, the 1^st^ layer cathode fractures at 3^rd^ pre‐notch in Model 8, while it is not valid in Model 7 (with coating connected). Besides, since Cu pre‐notch is larger than Al in Model 8, the gap between both sides of the fracture in 2^nd^ layer anode (gap‐An‐L_2_) is larger than in 1^st^ layer cathode (gap‐Ca‐L_1_), leaving room for the cathode to squeeze in. Then, the dislocation of the cathode happens, leading to the Al‐An contact (Figure 5c). In Model 7, the gap‐An‐L_2_ is smaller than the gap‐Ca‐L_1_, which reduces the possibility of the cathode being squeezed in the anode region and helps to avoid Al‐An contact (Figure 5c).

#### 3.3.2 Distribution Effect

First, Models 9 and 10 are established to investigate the effect of distribution density. The interval between adjacent pre‐notches (*s_notch_*) in Model 9 is half that in Model 6 with the distribution density marked as ‘2′ while the *s_notch_* in Model 10 is twice that in Model 6 with the distribution density marked as ‘0.5′ (Figure 5a). Note that for the condition where the distribution density keeps the same as Model 6, the distribution density is marked as ‘1′. Besides, Models 11 and 12 are established to explore the effect of the location difference between pre‐notches of Al and Cu. In Model 11, each pre‐notch of Al is more front with 0.1 mm closer to the loading center (*r* = 0) than that of Cu, while in Model 12, is 0.1 mm farther from the loading center (Figure 5a). Note that for the condition where pre‐notches of Al and Cu are aligned, the location difference is marked as ‘0′.

For the distribution density effect, in Model 10, the Al‐L_2_ contacts An‐L_2_, forming the Al‐An mode at the 1^st^ pre‐notch (of Model 10) (Figure 5d). The electrodes remain relatively intact within the vicinity of the 2^nd^ pre‐notch, far from the loading center (Figure 5d). Therefore, the Al‐L_2_ and An‐L_2_ within the vicinity of the 1^st^ pre‐notch are connected with the Ca‐Main and An‐Main, respectively, to form a stable Al‐An mode. Compared with Model 10, Model 9 has more fractures from the 3^rd^ to 5^th^ pre‐notch, where the Al‐An contact will not trigger stable ISCs (Figure 5d). The risky region in Model 9 is focused on the vicinity of the 6^th^ pre‐notch, where the Ca‐L_1_ and Cu‐L_1_ can contact each other to trigger the Ca‐Cu mode. The denser the pre‐notch distribution is, the more fractures are produced within the stress concentration region (from the 3^rd^ to 5^th^ pre‐notch in Model 9) where the maximum stress can reach 396.7 MPa (Figure 5d). The risky region will be farther from the loading center (where the stress is ≈80 MPa (Figure 5d)) to mitigate fracture behaviors, which can greatly reduce the risk of Al‐An mode.

For the location difference effect, in Model 11, with the pre‐notch of Al more forward, the fracture of the 1^st^ layer cathode will be more forward than that of the 2^nd^ layer anode within the vicinity of the 3^rd^ pre‐notch (Figure 5e). Then, the risk of contact between Al‐L_1_ (connected with Ca‐Main) and An‐L_2_ (connected with An‐Main) can be greatly reduced. The possible, stable ISC mode becomes the Ca‐Cu mode by the contact between Ca‐L_1_ and Cu‐L_1_ (Figure 5e). However, in Model 12, with the pre‐notch of Al less forward, the fracture of anodes becomes more forward than the fracture of the cathode within the vicinity of the 2nd pre‐notch. Then, the contact between the exposed Al‐L_1_ (connected with Ca‐Main) and An‐L_2_ (connected with An‐Main) becomes more likely (Figure 5e), and the stable Al‐An mode can be triggered inevitably. Note that the cathode with the pre‐notch of Al closer to the loading center in Model 11 will experience an easier fracture than in Model 12. Therefore, the 1^st^ layer cathode can fracture at the 3^rd^ pre‐notch in Model 11 while only fracturing at the 2^nd^ pre‐notch in Model 12 with the pre‐notch of Al farther away from the loading center.

With the results of the above models summarized (Figure 5b; with coordinates of points listed in Table S5, Supporting Information), three conditions can help the LIBs avoid the Al‐An mode after failure, where 1) *w_notch_* of Al is not smaller than *w_notch_* of Cu (the size difference is not smaller than 0); 2) the distribution of pre‐notch is dense (the distribution density is not smaller than 1); and 3) the pre‐notch of Al is not farther from the loading center than the pre‐notch of Cu. Note that in practical application, since the position of the loading point cannot be guaranteed, keeping the pre‐notches on Al and Cu aligned as many as possible is necessary.

## 4 Conclusion

In this study, first, the post‐mortem characterization of the deformed LIBs was conducted. Besides the disassembling, we obtained the section of the LIB with epoxy resin sealed by wire‐electrode cutting. Before the load drop exceeded 40% of the maximum value (defined as the battery failure), the LIB with 60% SOC only experienced failure of the separator when the Ca‐An mode was dominant. After the major mechanical failure of the cell, the Al‐An mode was confirmed based on the element distribution analysis on the section through the EDS. Besides, a locally refined mechanical model was established and accurately predicted the ISC behaviors under different loadings proved by experiments. The validated mechanical model supports revealing the relation between the stress‐driven ISCs and material failures. Based on the experimental ISC resistance (*R*_ISC_)‐stress curves, the *R*_ISC_ was obtained rationally and applied in the multiphysics model. The multiphysics model can accurately predict the electrothermal responses of the LIBs under different loadings, which supports the successful revelation of the mechanical‐electrical‐thermal coupling mechanism. Further, the SOC effect on the ISC mode after battery failure was discussed. The ISC mode under high SOC situations (≥ 30%) was Al‐An mode, different from the Al‐Cu mode at 0% SOC situation. The SOC effect on the failure behavior of anodes under indentation may contribute to this phenomenon. Finally, to improve the ISC mode after battery failure, fabricating surface notches on electrodes was selected as a representative method to mitigate the ISC consequence and optimal design guidance was provided. Results highlight the importance and mechanism of SOC dependency for the ISC behaviors and provide a powerful tool for designing next‐generation robust lithium‐ion batteries.

## 5 Methodology

### 5.1 Testing Sample Description

A commercial NCM (LiNi_x_Co_y_Mn_(1‐x‐y)_O_2_)/graphite pouch LIB was chosen as the sample (Table S1, Supporting Information). Inside the LIB, 14 layers of the anode (consisting of two layers of anode coatings (An) and one layer of the copper foil (Cu)), 13 layers of the cathode (consisting of two layers of cathode coating (Ca) and one layer of aluminum foil (Al)), and 28 layers of the separator with the thickness parameters given (Table S2, Supporting Information) constitute a jellyroll, in the sequence of separator‐anode‐separator‐cathode from the upper surface to the bottom surface of the LIB.

### 5.2 Cell Indentation Test

The steel ball indentation test can trigger highly controllable and repeatable ISCs of LIBs. A steel ball with a radius of 4 mm (*R*_ball_ = 4 mm) was placed on the center of the upper surface of the samples (Figure S1a, Supporting Information). The SUNS UTM5205X electric material testing machine equipped with an explosion‐proof box applied loadings on the LIBs with different SOCs (i.e., 0%, 30%, 60%, and 80%) at the speed of 1 mm min^−1^ with 50N as the preload. The tabs of the sample were connected to an Agilent 34972A Data Collector. The terminal voltage of LIBs was recorded at a frequency of 10 Hz. Two temperature measuring points 10 mm from the loading center (Figure S1a, Supporting Information) were also connected to the Agilent 34972A Data Collector with the same frequency. Then, the synchronous recording of the load (*F*), displacement (*d*), voltage (*U*), and temperature (*T*) of samples was achieved. The tests were repeated at least twice to ensure repeatability with the experimental results provided (Figures S6 and S7, Supporting Information).

### 5.3 Material Test

The components of the LIB include the battery cover, separator, cathode coating, Al, anode coating, and Cu. Basic mechanical experiments were carried out on each component to obtain mechanical parameters (Figure S1b, Supporting Information). The battery cover, Cu and Al were typical elastoplastic materials, and tensile tests were carried out. Note that the Cu and Al were obtained by being separated from the anode and cathode, respectively.^[^ ^9c^ ^]^ The samples for tensile tests were all cut into 5mm × 60 mm rectangles with a clamping area of 15 mm in length at both ends. The INSTRON 2386 material testing machine (with a range of 300 kN and accuracy of ±0.5% of the indicated value) was used for tensile loadings at a speed of 10 mm min^−1^ with results provided (Figure S8, Supporting Information). Besides, the out‐of‐plane compression test was carried out for the separator.^[^ ^13d^ ^]^ The separators were cut into 30mm × 30 mm squares. Considering the small thickness of the separator, 20 layers of the separator were stacked and then compressed by the INSTRON 2386 at a speed of 0.5 mm min^−1^ with 10N as the preload. Finally, the steel ball indentation tests were carried out to calibrate the cathode and anode coatings. The samples were obtained from the LIBs with different SOCs (i.e., 0%, 30%, and 60%) and cut into 30 mm × 30 mm squares. The steel ball with *R*_ball_ = 4 mm was placed on the middle of upper surface of 15‐layer cathode stacks and 15‐layer anode stacks respectively (Figure S1b, Supporting Information). The INSTRON 2386 was used for loading at a speed of 1 mm min^−1^ with 5N as the preload. The tests were repeated at least twice to ensure repeatability with experimental results provided (Figure S8, Supporting Information).

### 5.4 Charge–Discharge Test

The LIBs for indentation tests were charged to different SOCs (i.e., 0%, 30%, 60%, and 80%) after the full discharge by the BK6808AR cycler. Besides, the LIBs with full discharge were charged at various constant currents (CC) (i.e., 0.1C (capacity), 0.2C, 0.5C, and 1C rates) until the cut‐off voltage was 4.35 V separately. After that, the constant voltage (CV) charging method was applied to LIBs until the 0.25C cut‐off current. Then, the LIBs were discharged at corresponding rates (i.e., 0.1C, 0.2C, 0.5C, and 1C) until the cut‐off voltage of 2.8 V. The *U–t* curves were used to calibrate the battery model with the comparison results provided (Figure S10, Supporting Information).

### 5.5 Mechanical Modeling

*2D Mechanical Model for the Cell*: Based on the axial symmetry of the loading condition and material properties,^[^ ^6^, ^9^ ^]^ an axisymmetric 2D region around the steel ball within 10 mm was established (Figure S1c, Supporting Information). Considering the convergence, the geometric region for components from the cover to the 2^nd^‐layer cathode was divided based on their thickness (Table S2, Supporting Information), including the battery cover, 4‐layer of separators, 2‐layer of cathodes, and 2‐layer of anodes (including coatings and foils). The material parameters (Table S2, Supporting Information) calibrated by tests (Figure S9, Supporting Information) were assigned in each corresponding region. In the rest of the 2D region, the homogenized mechanical parameters of the jellyroll (Table S2, Supporting Information) were assigned. Both the steel ball moving down to squeeze the LIB and the fixed platform were built into the rigid body. A surface‐to‐surface contact method was applied among different components with a friction coefficient of 0.3. The approximate global mesh size of each component was set as 0.1 mm.

*Mechanical Models for Materials*: The battery cover was modeled using an elastoplastic approach, with parameters calibrated through tensile testing and summarized (Table S2, Supporting Information). The cathode and anode were each divided into a three‐layer structure comprising two‐layer coatings and a single‐layer foil, characterized by elastoplastic and crushable foam models, respectively. Parameters for each layer were calibrated through corresponding tensile and indentation tests. The cathode and anode coatings were built to ensure convergence based on shared nodes with their respective foils (Figure S1d, Supporting Information). The mechanical models for electrode indentation follow a similar approach as the 2D Mechanical Model for the cell, where a steel ball, 15 layers of anode/cathode, and a platform were established with a friction coefficient of 0.3. The jellyroll was modeled using an elastoplastic approach calibrated through cell indentation tests. The jellyroll indentation mechanical model was equivalent to the 2D Mechanical Model for the cell, but without refined regions. The approximate global mesh size for each component was set to 0.1 mm.

*Pre‐notches in 2D Mechanical Models*: The pre‐notches were set around the loading center (*r* = 0) within the 4 mm where the main deformation of the LIB occurs under steel ball indentation (with *R*_ball_ = 4 mm) (Figure S2, Supporting Information). The width of pre‐notch (*w_notch_*) on Al was set as 0.025 mm, and the depth (*d_notch_*) was set as 9um. The 1^st^ pre‐notch starts at *r* = 0.8 mm. The interval between adjacent pre‐notches (*s_notch_*) was set as 0.8 mm. These settings had been applied in the cathode‐Li metal half cells by the research^[^ ^23^ ^]^ where the cells with cathode pre‐notched experienced limited temperature rise after failure and owned the same cycling performance with reference cells. Similarly, the *w_notch_* of Cu, cathode coatings, and anode coatings was also set as 0.025 mm. The *d_notch_* of Cu changes to 3um to keep Cu foils connected. The *d_notch_* of coatings was the corresponding thickness of a single layer coating. The distribution of pre‐notches of Cu and coatings keeps the same with that of Al.

The abovementioned models were established based on the software ABAQUS with mechanical parameters provided (Table S2, Supporting Information).

### 5.6 Combining 2D Mechanical Model and 2D Multiphysics Model

The 2D Mechanical Model could predict the contact status between electrodes, including the ISC mode, ISC location, and contact pressure, at any time during indentation. The 2D Multiphysics Model was established for predicting the electrothermal responses of the LIB after being loaded to that time based on the software COMSOL Multiphysics.^[^ ^11^ ^]^ In this research, the 2D Mechanical Model and 2D Multiphysics Model were combined at three critical points (Points 1–3 in Figure 1), enough to reveal the mechanical‐electrical‐thermal coupling mechanism (Section 2.3). The specific combining method is as follows:

Step 1. Based on the 2D Mechanical Model, coordinates of both sides at the contact of cathode and anode can be obtained (Figure S3a, Supporting Information), including the points a‐b for the situation where the LIB was loaded to Point 1 (Point‐1‐situation), the points c‐d for Point‐2‐situation and points e‐f for Point‐3‐situation with specific values provided (Figure S3c, Supporting Information);
Step 2. The ISC region preset in the ISC module in 2D Multiphysics Model could be determined. The geometry of the 2D Multiphysics Model was an axisymmetric 2D region with a length of half the width of the battery (Figure S3b, Supporting Information). Along the direction of the *z*‐axis, the 2D region was divided based on the thickness of each component layer, including the battery cover, separator, anode, and cathode (consisting of two‐layer coating and one‐layer foil). Then, the ISC region was set within the An‐L_1_, Sep‐L_1_, and Ca‐L_1_ at Points 1 and 2 and within the Ca‐L_1_, Sep‐L_2_, and An‐L_2_ at Point 3. Along the direction of *r* axis, the width range of the ISC region is consistent with the *r* coordinate of each point (a–f) (Figure S3b, Supporting Information);
Step 3. The ISC conductivity κ_*ISC*_ for the ISC region could be obtained by the law of resistance (Equation 1) with the method specifically introduced (in Section 2.3).

### 5.7 Multiphysics Modeling

The 2D Multiphysics Model consists of a mechanical module, ISC module, battery module, heat module, and thermal runaway module (Figure S4a, Supporting Information).

The mechanical module calculates the deformation of the whole cell under the steel ball indentation, providing the geometric boundary for other modules. The governing equation of the mechanical model follows Newton's second law:

(3) $$\rho \frac{\partial^{2}u}{\partial t^{2}}=\nabla \left(FS\right)+F_{V}$$

where ***u*** is the displacement field, ρ is density, *S* is the Piola–Kirchhoff stress tensor, *F* is the deformation gradient, and *F_V_* is the body force. Considering the stringent convergence requirement, the battery is characterized as a homogeneous jellyroll with mechanical parameters provided (Table S2, Supporting Information). The steel ball and platform were also built into the rigid body same. The contact method among the steel ball, platform, and the battery was set as a penalty with automatic factor control.

The ISC module with the conductivity κ of each region provided (Table S2, Supporting Information) computes the current distribution *i* and the ISC‐induced heat generation *Q*_joule_, based on the distribution of conductivity κ and the electric field between foils $\vec{E}$ as follows:

(4) $$\vec{i}\;=\;\kappa \vec{\nabla }\phi$$

(5) $$\vec{E}=-\vec{\nabla }\phi$$

(6) $$I_{ISC}=\underset{\Omega }{\;\int \int \;}\vec{i}d\Omega$$

(7) $$Q_{joule}=Q_{ISC}+Q_{cell}=\frac{\vec{i}\cdot \vec{i}}{\kappa }$$

where ϕ is potential, Ω is the boundary for all layers of the separator, *Q_ISC_* and *Q_cell_* are the heat sources from *R*_ISC_ and *R*_cell_ *_,_* respectively. The *I*_ISC_ is transferred to the battery module as the boundary condition. The terminal voltage of the LIB calculated by the battery module is assigned to the Al foils and the voltage of the Cu foils is set as 0, which determines the $\vec{E}$.

The 1D battery model, including one‐side cathode coating, separator, and one‐side anode coating, was established and used to predict the terminal voltage of the LIB. The results from the above charge–discharge test have validated the battery model with the parameters given (Table S3, Supporting Information).

The heat module with thermal parameters provided (Table S2, Supporting Information) was used to calculate temperature distribution. The equations are as follows:

(8) $$\rho C_{p}\left(\frac{\partial T}{\partial t}+u\nabla T\right)+\nabla q=\;Q$$

(9) q=−k∇T

(10) $$Q\;=\;Q_{joule}+Q_{TR}$$

where ρ and *C_p_*, *k* is the density, heat capacity, and thermal conductivity of each component, respectively, *u* is the displacement field computed by the mechanical module, *Q* is the total heat sources, and *Q_TR_* is the heat source from the thermal runaway module (W m^−3^).

The thermal runaway module considers three main chemical reactions: SEI decomposition, cathode‐electrolyte, and anode‐electrolyte reactions for reasonable simplification. *Q_TR_* can be calculated by:^[^ ^24^ ^]^

(11) $$Q_{x}\left(t\right)=m_{TRx}H_{x}c_{x}^{n_{x}}A_{x}\exp\left(-\frac{E_{x}}{RT}\right)$$

(12) $$Q_{TR}=Q_{s}+Q_{a}+Q_{c}$$

where *m_TRx_* is the mass participating in each reaction *x*, *A_x_* and *E_x_* are the pre‐exponential factor and activation energy, respectively, *c_x_* is the normalized concentration of each reaction *x*, *R* is the gas constant, and the subscript *x* will be *c*, *a*, and *s* representing the cathode‐electrolyte reaction, anode‐electrolyte reaction, and SEI decomposition, respectively.

The above 2D Multiphysics Model was established based on the software COMSOL Multiphysics with a more detailed simulation method that can be found in the previous research.^[^ ^9a^ ^]^

## Conflict of Interest

The authors declare no conflict of interest.

## Supporting information

Supporting Information

## Data Availability

The data that support the findings of this study are available in the supplementary material of this article.
