# Supplementary material for: Understanding of Stress‐Driven Internal Short Circuit Mechanisms in Lithium‐Ion Batteries with High SOCs
Source: Adv Sci (Weinh). 2023 Aug 9;10(29):2302496. doi: 10.1002/advs.202302496 (PMC10582443; doi:10.1002/advs.202302496)
Supplement: Supplementary file 1 — Supporting Information [file ADVS-10-2302496-s001.pdf]

ADVANCED SCIENCE
Open Access

Supporting Information

for *Adv. Sci.*, DOI 10.1002/advs.202302496

Understanding of Stress-Driven Internal Short Circuit Mechanisms in Lithium-Ion Batteries with High SOCs

*Xudong Duan, Jiani Li, Yikai Jia, Xiang Gao, Lubing Wang and Jun Xu**

Supporting Materials

Understanding of Stress-driven Internal Short Circuit Mechanisms in Lithium-ion Batteries with high SOCs

Xudong Duan[a], Jiani Li[b, c], Yikai Jia[d], Xiang Gao[b, c], Lubing Wang[e], Jun Xu[b, c ,f*]

[a]*Department of Automotive Engineering, School of Transportation Science and Engineering, Beihang University, Beijing 100191, China*

[b]*Department of Mechanical Engineering and Engineering Science, The University of North Carolina at Charlotte, Charlotte, NC 28223, USA.*

[c]*Vehicle Energy & Safety Laboratory (VESL), North Carolina Motorsports and Automotive Research Center, The University of North Carolina at Charlotte, Charlotte, NC 28223, USA.*

[d]*School of Civil Aviation, Northwestern Polytechnical University, Xi'an, Shaanxi, 710072, China*

[e]*Key Laboratory Impact & Safety Engineering, Ministry of Education, Ningbo University, Ningbo 315211, Zhejiang, China*

[f]*School of Data Science, North Carolina Motorsports and Automotive Research Center, The University of North Carolina at Charlotte, Charlotte, NC 28223, USA.*

[*]Corresponding author: Prof. Jun Xu at jun.xu@uncc.edu; Tel: (704)-687-8240; Fax: (704)-687-8240

We employed steel-ball indentation tests on an NCM ($LiNi_xCo_yMn_{(1-x-y)}O_2$)/graphite pouch LIB (Table S1) to identify ISC modes with basic information listed as follows:

Table S1. Basic information about battery samples

Parameters	Value
Nominal capacity	3.577 Ah
Charging/Discharging cutoff voltage	4.35 V/2.8 V
Length/width/height	97/58/4.5 mm
Internal resistance	50 mΩ

To produce a highly repeatable and stable triggering of ISCs, we designed a steel ball indentation to reliably trigger the ISCs. A locally detailed mechanical model was established to predict the mechanical response of the LIB under the steel-ball indentation. The material tests were also carried out to support material mechanical models (Figure S1).

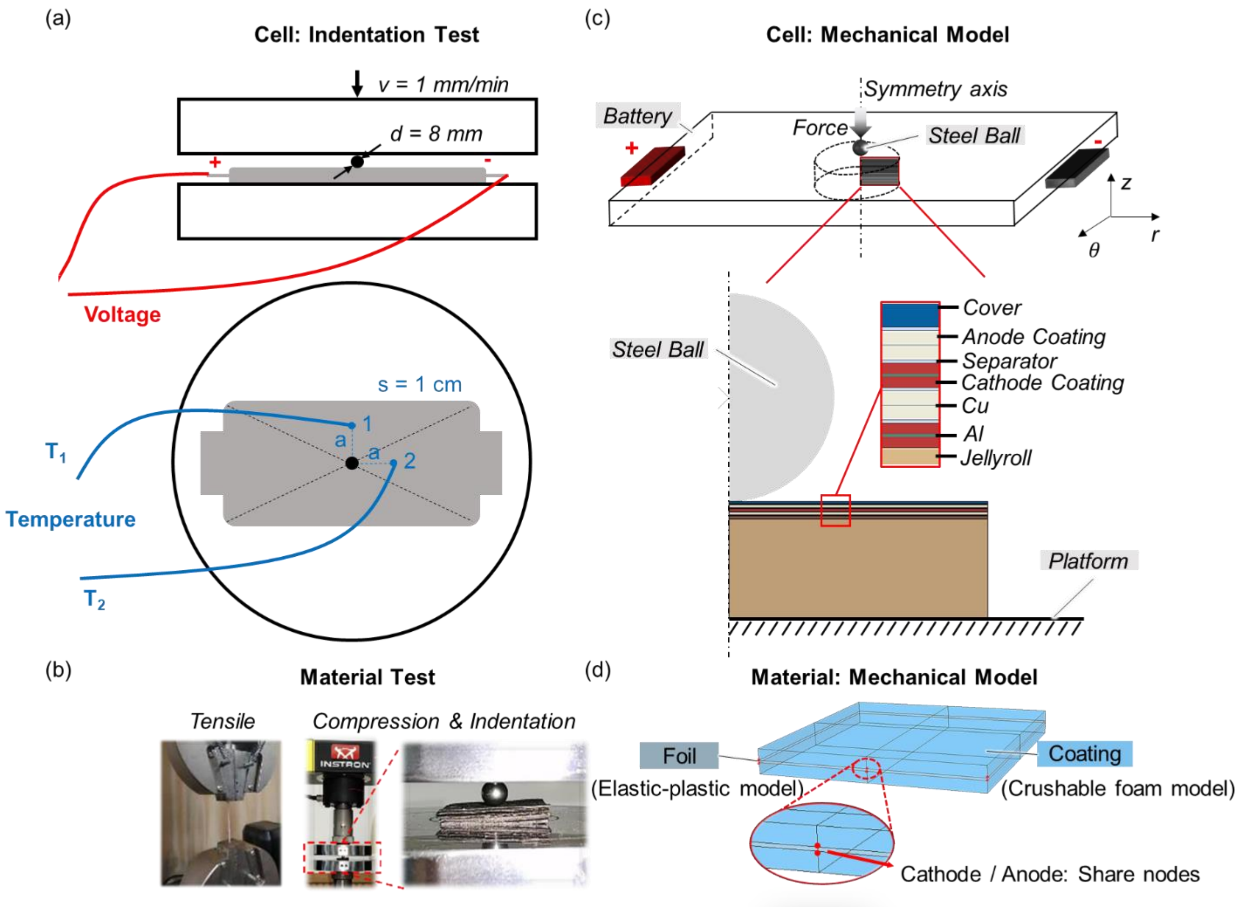

Figure S1. Experimental settings for (a) indentation tests of the LIBs; and (b) internal components of the LIB. Mechanical modeling settings for (c) the cell; and (d) the electrodes.

To improve the ISC mode after battery failure, setting surface notches on electrodes was selected as a representative method with the effect discussed based on the 2D Mechanical Model with the geometry of surface notches introduced (Figure S2).

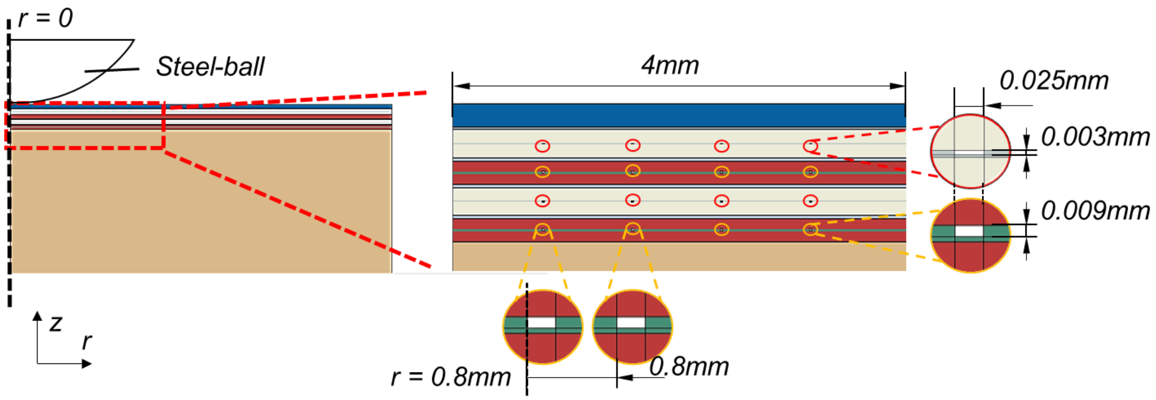

Figure S2. Geometry of the pre-notch set in Model 6.

In this research, the 2D Mechanical Model is established based on the software ABAQUS. The 2D Multiphysics Model is established based on the software COMSOL Multiphysics [1]. The 2D Mechanical Model and 2D Multiphysics Model are combined at three critical points (Points 1~3 in Figure 1) with specific combining method summarized (Figure S3).

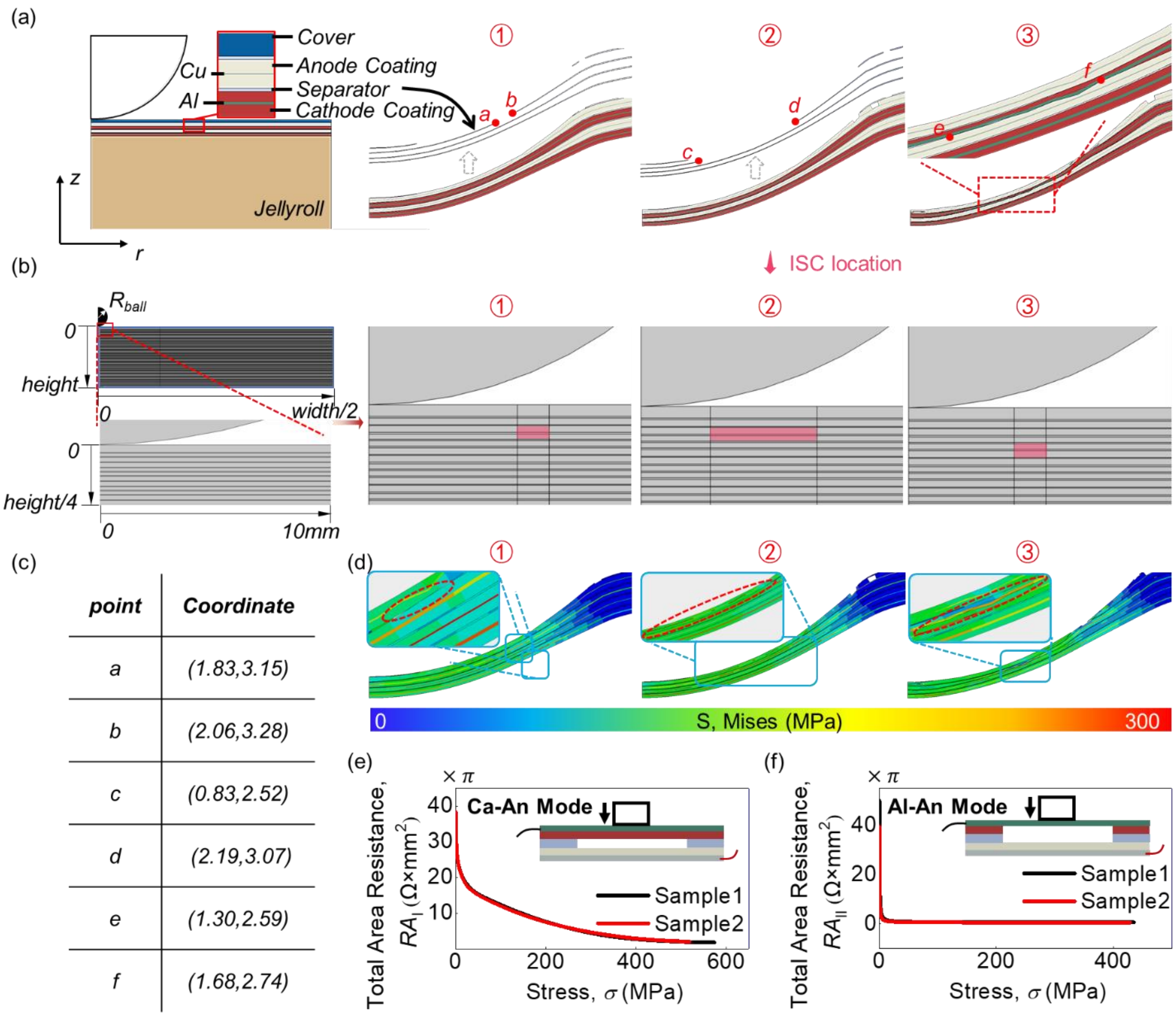

Figure S3. Method of combining the 2D Mechanical Model and 2D Multiphysics Model. (a) the ISC locations at three critical Points predicted by the 2D Mechanical Model; (b) the pre-set ISC locations set in the 2D Multiphysics Model at three critical Points; (c) the coordinates of the ISC locations at three critical Points predicted by the 2D Mechanical Model; (d) the stress distribution at three critical Points computed by the 2D

Mechanical Model; the experimental total area resistance (RA)-stress (σ) curves of the (e) Ca-An mode and (f) Al-An mode.

The 2D Multiphysics Model consists of a mechanical module, ISC module, battery module, heat module, and thermal runaway module with the modeling method provided (Figure S4).

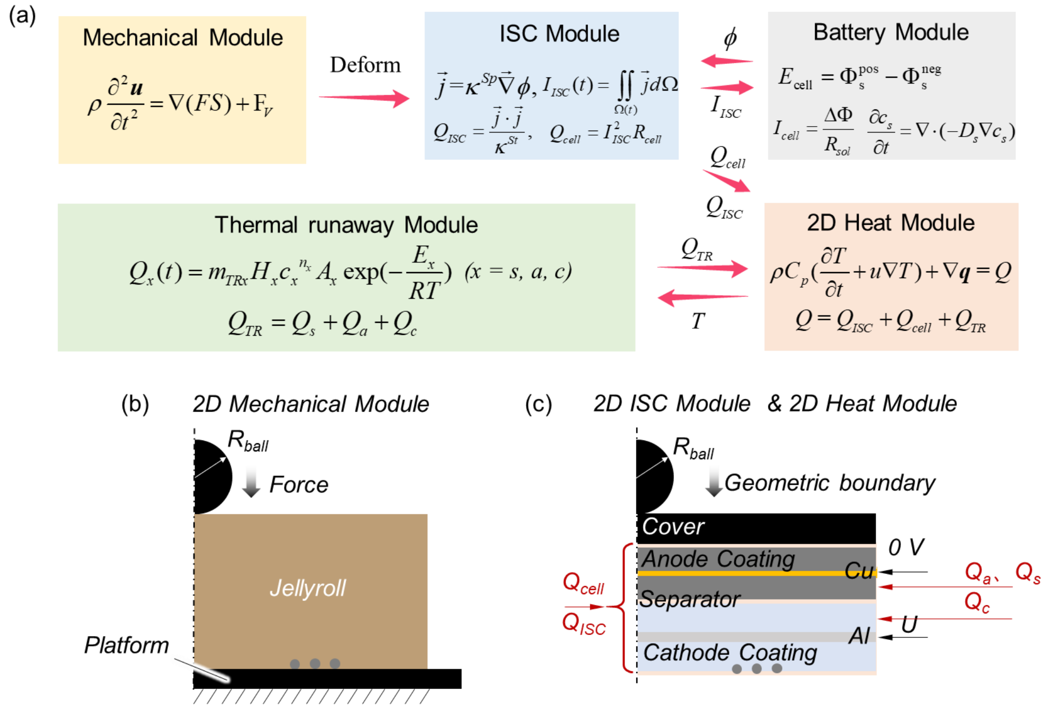

Figure S4. (a) Coupling strategy and schematic algorithms for modules, b) schematics of the geometry of models and boundary conditions for c) 2D mechanical module and d) 2D ISC and heat modules.

The geometric, mechanical, electrical and thermal parameters for models are summarized as follows (Table S2).

Table S2. Parameters of components for models.

				Plastic parameters			Failure parameters			Thermal parameters	
Components	Thickness (mm)	Modulus (MPa)	Poisson ratio	Yield stress (MPa)	Crushable foam		Damage initiation strain	Damage evolution	Conductivity (S/m)	Thermal conductivity (W/(m•K))	Heat capacity (J/(kg•K))
					k	k_t					
Battery cover	0.11	4240	0.3	8.00	/	/	/	/	3.8e7 [2]	/	/
Jellyroll	/	500	0.15	0.1	/	/	/	/	/	/	/
Cathode-foil	0.013	21400	0.3	30.82	/	/	0.35	0.001	3.8e7 [2]		
Cathode-coating	0.051	6000	0	/	2.3	0.05	0.5	0.54	100 [2]		
Anode-foil	0.008	22600	0.3	126.05	/	/	0.55	0.001	6.0e7 [2]	238	900
Anode-coating	0.0635	200	0.15	/	2.3	0.05	0.4	0.145	270 [2]		
Separator	0.015	2000	0.3	/	2.3	0.05	1	0.04	1e-6 [2]		

The detailed electrochemical parameters for the 1D battery model are summarized (Table S3).

Table S3. Electrochemical parameters of the battery.

**Parameters**	**Value**	**Source**
Positive stoichiometry at full charge	1	Calibrated
Positive stoichiometry at full discharge	0	Calibrated
Negative stoichiometry at full charge	0.96	Calibrated
Negative stoichiometry at full discharge	0	Calibrated
OCV	curves	Figure S5 [3]
Diffusion coefficient in cathode	1.8E-14 m^2/s	Calibrated
Diffusion coefficient in anode	4E-14 m^2/s	Calibrated
Positive conductivity	100 S/m	[3a]
Negative conductivity	100 S/m	[3b]
Positive reaction rate coefficient, k_{pos}	5e-10 m/s	Calibrated
Negative exchange current density, k_{neg}	2e-11 m/s	Calibrated
Maximum concentration of cathode	49000 mol/m^3	[3a]
Maximum concentration of anode	31507 mol/m^3	[2, 3b]
Positive porosity	0.4	Provided
Negative porosity	0.4	Provided

Separator porosity	0.39	Provided

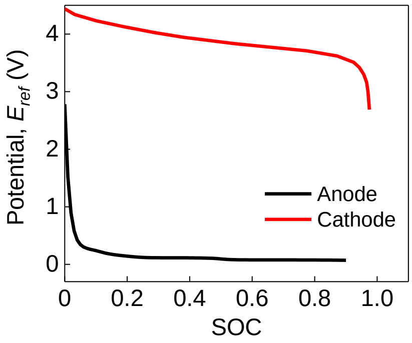

Figure S5. The thermodynamic equilibrium potential of electrodes.

The thermal parameters are used for calculating thermal runaway reaction heat sources with parameters referred to the research [4].

Table S4. Parameters for thermal runaway model

Parameters	Value
A_a	4.34e10 min^{-1}
A_c	6.9e10 min^{-1}
E_a	1.06e5 J mol^{-1}
E_c	1.277e5 J/mol
A_s	6.3623e9 s^-1
E_s	1.0960e5 J/mol
n_s	5.5
n_a	1
n_c	1
H_a	1712 J/g
H_c	1000 J/g
H_s	578.7 J/g
c_{a0}	0.75
c_{c0}	0.75
c_{s0}	0.15

The LIBs with 60% SOCs were squeezed by a steel ball with a radius of 4mm (R_{ball}=4mm) to trigger ISCs. LIBs were loaded to each critical point (Points 1~3) and the U and T of LIBs remained recorded after samples were unloaded. Then, the effect

of ISCs (electrothermal response of LIBs) triggered by different stresses was experimentally investigated (Figure S6).

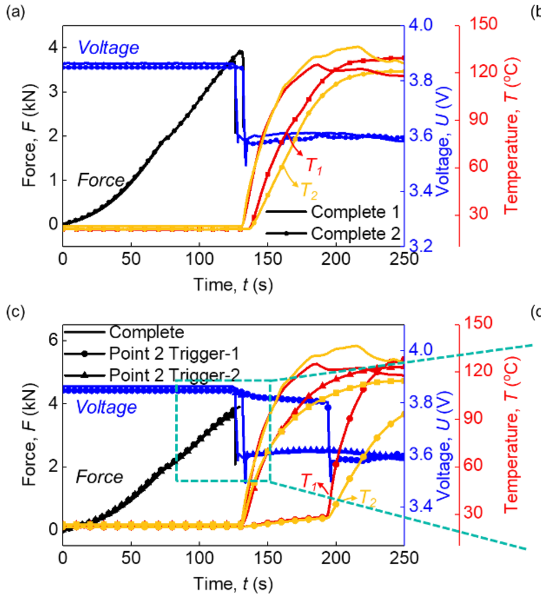

Figure S6. Experimental F-, U-, and T-t curves from steel-ball indentation for the LIBs with 60% SOC being loaded until (a) Point 3 (loaded completely); (b) Point 1; (c) Point 2 and (d) the enlarged view of (c) within t from 110s to 140s.

The LIBs with different SOCs (including 0%, 30%, 60% and 80%) were squeezed by the steel ball with results provided (Figure S7). Note that the temperature from measuring points 1 and 2 was averaged for each repeated test to display.

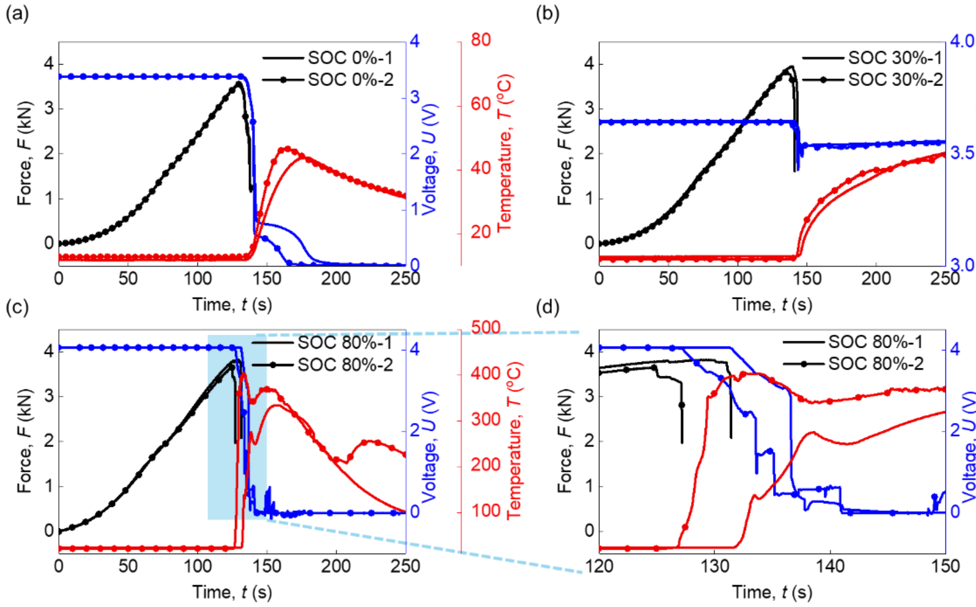

Figure S7. Experimental *F*-,*U*-, and *T*-*t* curves from steel-ball indentation for the LIBs with (a) 0% SOC; (b) 30% SOC; (c) 80% SOC (d) the enlarged view of (c) within *t* from 120s to 150s.

The components of the LIB including the battery cover, separator, cathode coating, Al, anode coating and Cu were tested by basic mechanical experiments with results provided (Figure S8).

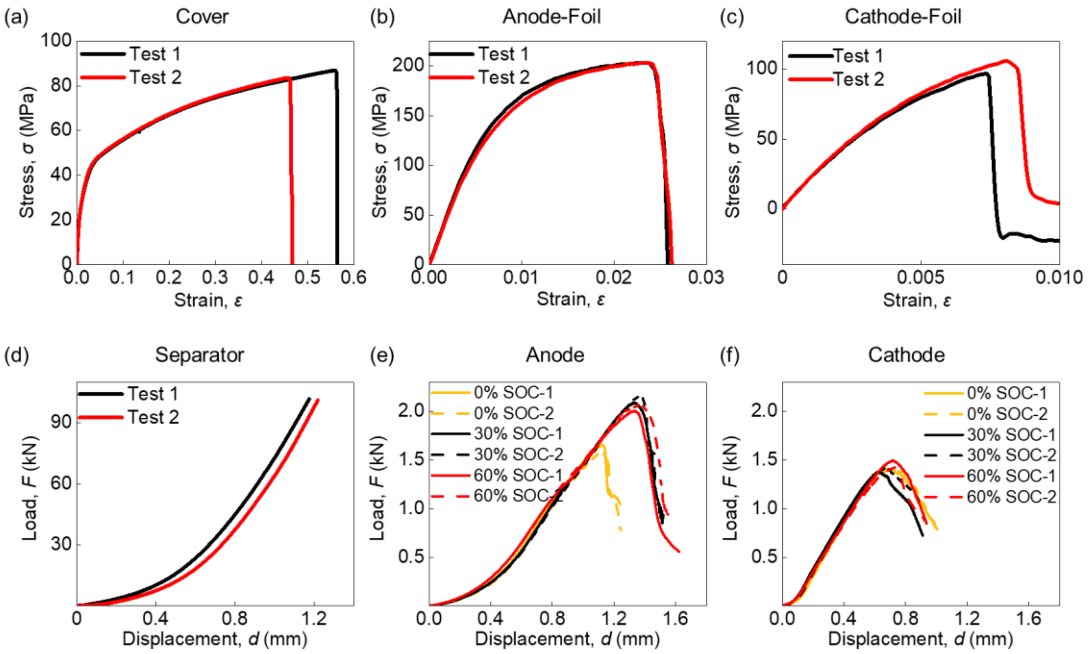

Figure S8. Experimental stress(σ)-strain(ε) curves from the (a) battery cover under tensile tests; (b) anode-foil under tensile tests; (c) cathode-foil under tensile tests; (d) separator under compression tests; (e) anode with different SOCs under indentation tests and (f) cathode with different SOCs under indentation tests.

Each component of the LIB (including the battery cover, separator, cathode coating, Al, anode coating and Cu) has the corresponding mechanical model under the material testing condition for calibrating mechanical parameters with comparison results summarized (Figure S9). Note that the failure of foils were not calibrated by tensile conditions but by the electrode indentation tests since the samples always had defects after being cut leading to early failure of electrodes [5].

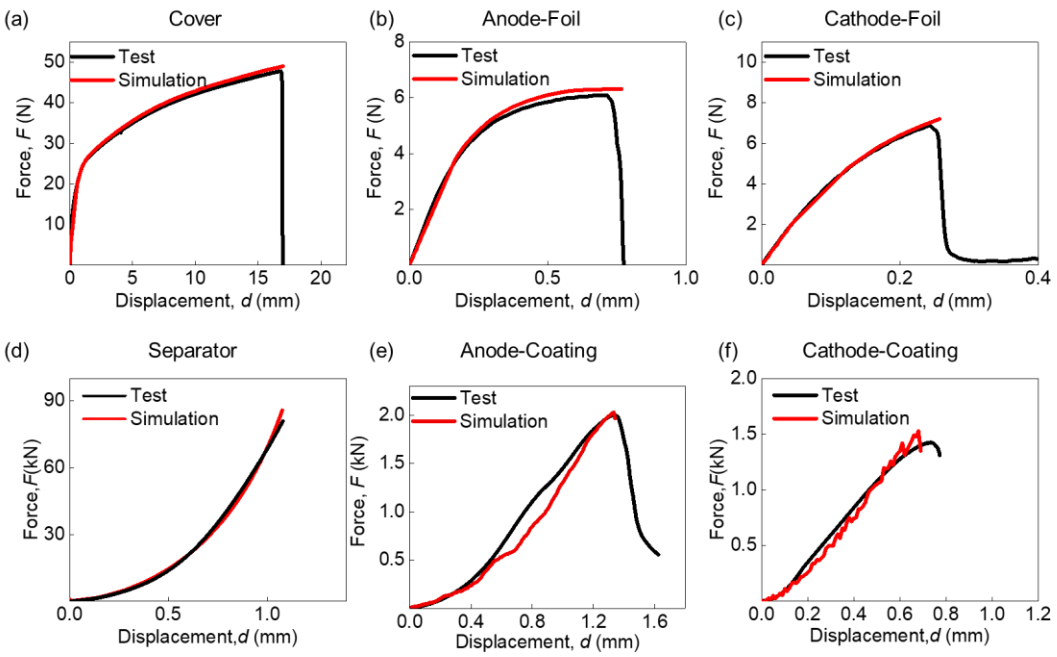

Figure S9. The *F-d* curves from the experiment and mechanical model of (a) battery cover under the tensile condition; (b) anode-foil under the tensile condition; (c) cathode-foil under the tensile condition; (d) separator under the compressive condition; (e) anode with 60% SOC under indentation condition and (f) cathode with 60% SOC under indentation condition.

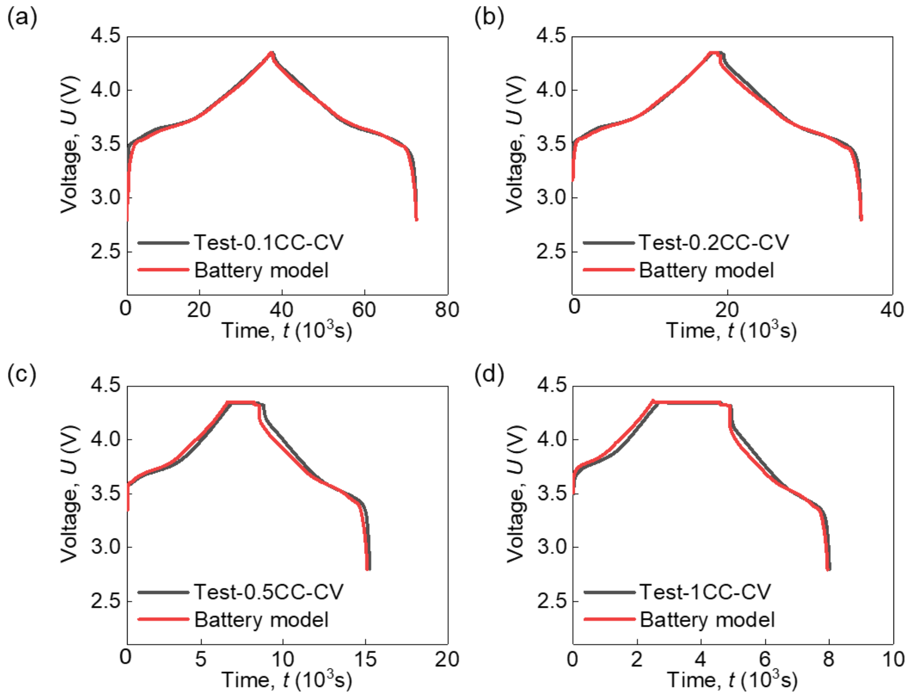

Figure S10. *U-t* curves from experiments and the battery model under (a) 0.1C constant charging (CC)-CV (constant voltage)-0.1C-CD (constant discharge) condition; (b) 0.2CC-CV-0.2C-CD; (c) 0.5CC-CV-0.5C-CD; (d) 1CC-CV-1C-CD;

The parametric study on size and distribution of pre-notches was carried out with supplementary information provided (Figure S11).

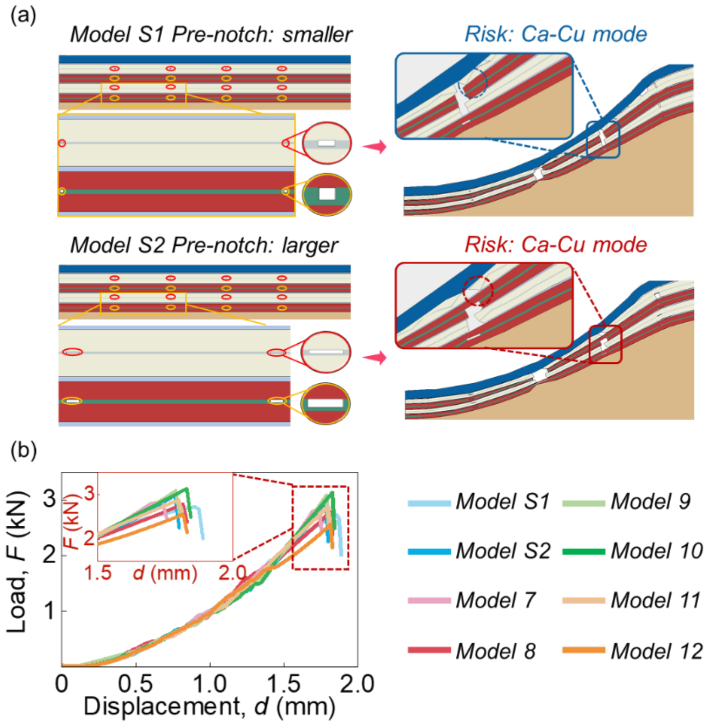

Figure S11. (a) The arrangement of pre-notches on Models S1-S2 and the failure morphology after the battery failure based on the 2D Mechanical Model; and (b) *F*-*d* curves of Models S1-S2 and 7-12.

Table S5. Coordinates of points for Figure 5b

Model No.	Size Difference	Distribution Density	Location Difference	ISC Mode Risk
6	0	0	0	Ca-Cu
7	1	0	0	Ca-Cu
8	-1	0	0	Al-An
9	0	1	0	Ca-Cu
10	0	-1	0	Al-An
11	0	0	0.1	Ca-Cu
12	0	0	-0.1	Al-An
S1	0	0	0	Ca-Cu
S2	0	0	0	Ca-Cu

Reference

[1] B. Liu, Y. Jia, J. Li, S. Yin, C. Yuan, Z. Hu, L. Wang, Y. Li, J. Xu, *Journal of Materials Chemistry A* **2018**, 6, 21475.

[2] H. Li, B. Liu, D. Zhou, C. Zhang, *Journal of The Electrochemical Society* **2020**, 167.

[3] a) J. B. Siegel, A. G. Stefanopoulou, P. Hagans, Y. Ding, D. Gorsich, *Journal of The Electrochemical Society* **2013**, 160, A1031; b) M. Doyle, Y. Fuentes, *Journal of The Electrochemical Society* **2003**, 150, A706.

[4] X. Duan, H. Wang, Y. Jia, L. Wang, B. Liu, J. Xu, *Energy Storage Materials* **2022**, 45, 667.

[5] B. Liu, X. Duan, C. Yuan, L. Wang, J. Li, D. P. Finegan, B. Feng, J. Xu, *Journal of Materials Chemistry A* **2021**, 9, 7102.
